# Research hotspots and evolution trends of human amniotic fluid stem cells: A cite space bibliometric analysis based on WoSCC and PubMed

**DOI:** 10.1097/MD.0000000000051013

**Published:** 2026-10-16

**Authors:** Huanying Lou, Zheng Wang, Shuai Zhang, Feng Wang, Qiang Feng, Yi Li

**Affiliations:** a Prenatal Diagnostic Laboratory, The Affiliated Taian City Central Hospital of Qingdao University, Taian, China; b Department of Medical Laboratory Science, The Affiliated Taian City Central Hospital of Qingdao University, Taian, China.

**Keywords:** bibliometrics, CiteSpace, extracellular vesicles, human amniotic fluid stem cells, knowledge graphs, research hotspots

## Abstract

**Objective:**

To systematically review the research hotspots, knowledge structure, and evolution trends in the field of human amniotic fluid stem cells (hAFSCs).

**Methods:**

A bibliometric approach was employed using CiteSpace 6.2.R4 software, with the Web of Science Core Collection (WoSCC, 2006–2026, including 601 original research papers) and PubMed (1984–2024, including 960 publications) as data sources. Standardized, reproducible search strategies (including synonyms, abbreviations, MeSH terms, and Boolean operators) were developed for each database, and literature screening followed the PRISMA 2020 guidelines. Using the WoSCC dataset as the core, we generated comprehensive visualizations covering author collaboration networks, countries, institutions, keyword co-occurrence, clustering, timelines, time zones, and emerging terms; the PubMed data were used for comparison of long-term publication trends.

**Results:**

The global publication volume in the field of hAFSCs has shown an overall upward trend, which can be divided into 3 phases: exploratory inception (1984–2006), rapid growth (2007–2013), and stable deepening (2014 to present). The United States (177 articles) and Italy (132 articles) form the dual engines of international collaboration, while China ranks third in publication volume (75 articles) but exhibits relatively weak international collaboration (centrality 0.02). The evolution of research themes is clear: from 2006 to 2009, the focus was on cell isolation, characterization, and in vitro culture; from 2010 to 2015, the scope expanded to multipotent differentiation, animal models, and the treatment of fetal diseases; and since 2018, exosomes/extracellular vesicles have emerged as the most active frontier with the highest emergence intensity (7.37).

**Conclusion:**

Bibliometric analysis indicates that research on hAFSCs has evolved from basic biological characterization into a comprehensive field encompassing multipotent differentiation, tissue engineering, and exosome regulation. Exosomes/extracellular vesicles and targeted repair of organ damage are currently the primary areas of focus. This study is a descriptive analysis; all conclusions are based on publication trend statistics and do not involve validation of clinical translational efficacy.

## 1. Introduction

Human amniotic fluid stem cells (hAFSCs) are mesenchymal stem cells derived from fetal amniotic fluid; they account for approximately 1% of the total suspended cells in amniotic fluid and stably express the stem cell marker CD117 (c-kit).^[1]^ In 2003, Prusa’s team first demonstrated the presence of multipotent stem cells in amniotic fluid. Since then, hAFSCs have become a key research focus in the field of regenerative medicine due to their advantages, including easy access to source material, excellent proliferation and differentiation capabilities, the absence of ethical controversies associated with embryonic stem cells, and a low risk of tumorigenesis.^[2,3]^ Knowledge graphs are an interdisciplinary analytical tool that integrates applied mathematics, computer science, information science, and scientometrics; they can visually represent the development trajectory, core hotspots, and cutting-edge directions of a discipline through visual graphs.^[4–7]^ CiteSpace is currently the most widely used knowledge mapping tool in the medical field, leveraging data such as authors, institutions, keywords, and citations to uncover patterns of disciplinary evolution.^[8]^ As the standard citation database in the field of bibliometrics, WoSCC provides comprehensive data on author/institutional collaborations and disciplinary classifications, enabling multidimensional CiteSpace visualization analyses at the national, institutional, keyword, and emerging term levels. However, this database has limited coverage of early literature prior to 2006 the concept of hAFSCs was not formally proposed until 2003, and the sample size of original papers included in WoSCC between 2003 and 2005 was extremely low, making it impossible to construct stable collaboration networks and evolutionary maps. PubMed, on the other hand, excels in indexing biomedical abstracts and can supplement the early, scattered literature missing from WoSCC between 1984 and 2005, thereby fully presenting the field’s development trajectory from its inception to maturity. Therefore, this study established a dual-database retrieval framework centered on WoSCC and supplemented by PubMed: using the 601 original research papers from WoSCC (2006–2026) as the core dataset, we generated comprehensive visualizations across all dimensions, including author, country, and institutional collaboration networks, as well as keyword co-occurrence, clustering, timelines, time zones, and emerging terms. The 960 publications from PubMed (1984–2024) are used solely for time-series comparisons of annual publication volumes and are not included in the knowledge graph construction. This approach enables a multidimensional visual analysis of the global research landscape for hAFSCs, objectively mapping the field’s developmental trajectory and identifying potential research directions.

## 2. Methods

### 2.1. Data sources and search strategy

To balance the standardization of bibliometric analysis with comprehensive coverage of early literature in the field, this study conducted a collaborative search using 2 major databases: WoSCC and PubMed. Standardized, reproducible search queries were developed for both databases, and all search statements, time periods, and exclusion criteria were fully documented.This study does not require ethical approval as all data were obtained from publicly available databases and do not involve human subjects or personal privacy.

#### 2.1.1. WoSCC search strategy

The search period is January 2006 through July 2026. The search fields are Topic (title, abstract, keywords); the document type is restricted to Article; and the language is restricted to English. Search query: Topic TS=(“human amniotic fluid stem cell”) OR TS=(“human AFSC”) OR TS=(“human amniotic fluid-derived stem cell”) OR TS=(“human amniotic fluid mesenchymal stem cell”) OR TS=(“human amniotic fluid mesenchymal stromal cell”) OR TS=(“hAFSC”) OR TS=(“hAF-MSC”). After deduplication and screening, a total of 601 articles were ultimately included.

#### 2.1.2. PubMed search strategy

The search period was January 1984 through May 2024. The search was conducted in a single session in May 2024; the original results were not updated subsequently and were retained in their entirety for comparison of longitudinal trends. The search combined MeSH terms with synonyms from titles and abstracts, and was restricted to “Humans” as the study population throughout. Search Query: ((“Amniotic Fluid”[MeSH Terms]) AND (“Stem Cells”[MeSH Terms]) AND Humans [MeSH Terms]) OR ((“human amniotic fluid stem cell”[Title/Abstract] OR “human AFSC”[Title/Abstract] OR “hAFSC”[Title/Abstract] OR “amniotic fluid-derived stem cell”[Title/Abstract] OR “amniotic fluid mesenchymal stem cell”[Title/Abstract]) AND Humans [MeSH Terms]). The initial search yielded 962 records. After excluding 2 invalid studies (1 duplicate entry and 1 study involving only animal experiments), 960 studies were ultimately included. These were used solely for comparing publication timelines and were not included in the creation of the network diagram.

#### 2.1.3. Additional database selection criteria

Databases such as Scopus, Dimensions, and Embase were not included because they overlap significantly with the WoSCC literature. Including them would substantially increase the screening workload, and the current combination of 2 databases already meets the analysis requirements.

### 2.2. Literature screening process and inclusion and exclusion criteria

The literature screening for this study strictly adhered to the guidelines of the PRISMA 2020 Statement, and a standardized flowchart was created (Fig. 1), which fully documents the number of articles at each stage including searches across 2 databases, deduplication, initial screening, and rescreening as well as the reasons for exclusion.

**Figure 1.**
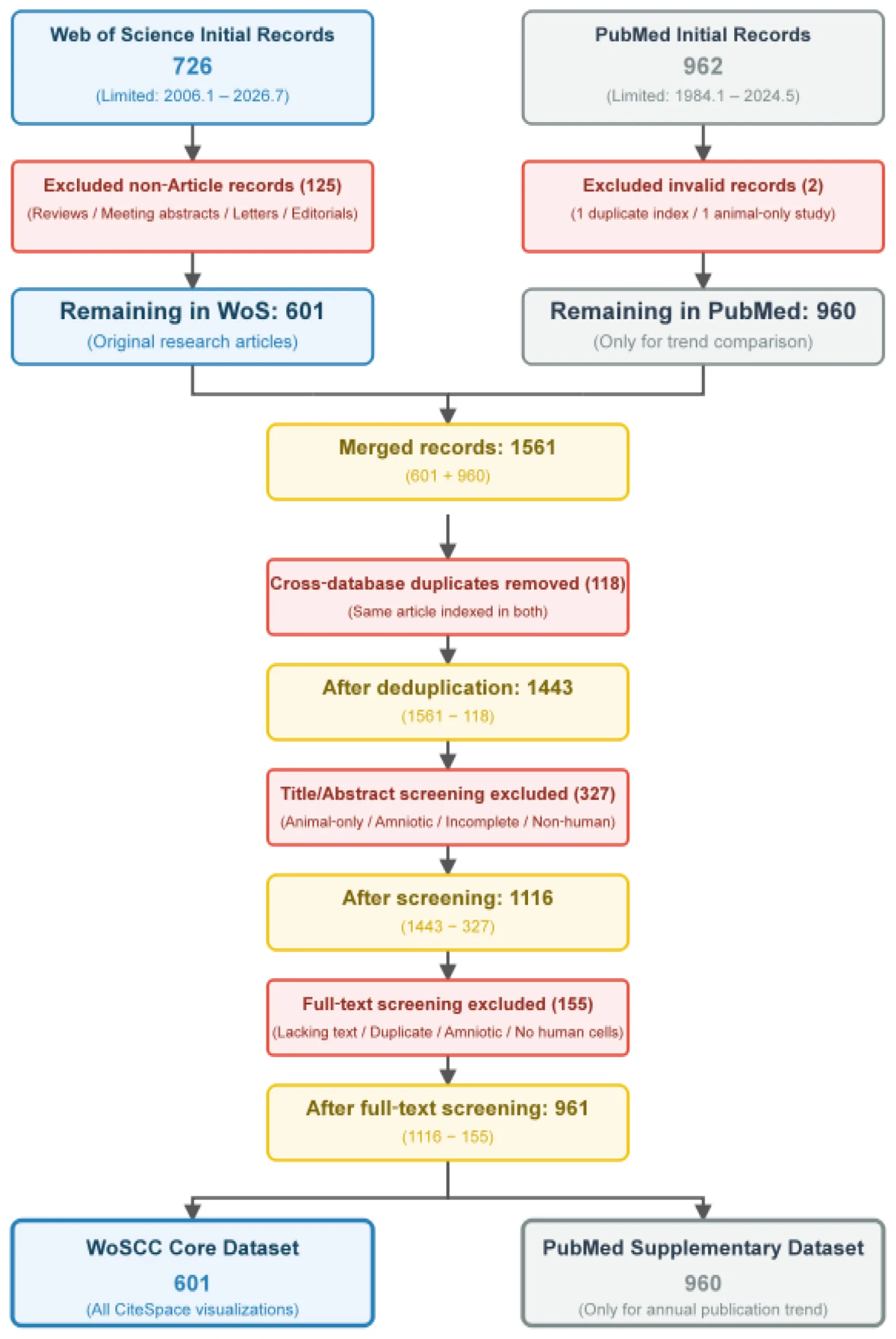
PRISMA 2020 flow diagram for dual-database literature search. WoSCC = Web of Science Core Collection. PRISMA = Preferred Reporting Items for Systematic Reviews and Meta-Analyses.

Article inclusion criteria: The study subjects are human amniotic fluid-derived stem cells; The full text is available in English; and WoSCC includes only original experimental articles (Article), while PubMed includes all document types and is used solely for comparing publication trends.

Article exclusion criteria: Studies involving only animal experiments, with no human cell data; Non-original literature such as reviews, conference abstracts, case reports, and editorial commentaries; Duplicate publications or records with incomplete bibliographic information that cannot be imported into the software; and Studies using amniotic stem cells (which differ from amniotic fluid stem cells in concept and research focus).

### 2.3. Analytical tools and parameter settings

We used Excel to create a line chart comparing annual publications across the 2 databases. Using CiteSpace 6.2.R4 software and based on the WoSCC dataset of 601 publications, we completed the creation of all visualization maps. A complete explanation of the software parameter settings and selection criteria is provided below:

#### 2.3.1. Time slices

2006 to 2026, with each slice covering a 1-year period, consistent with the WoSCC search timeframe.

#### 2.3.2. Node selection

The top 50 nodes by frequency are selected from each time slice. This threshold balances the completeness of the graph information with the readability of the visualization and is a standard setting for similar metric papers in the stem cell field.

The g-index (*k* = 25) is a metric for evaluating research performance. While the sheer number of publications merely reflects output volume, the g-index balances both publication volume and citation impact, providing a more objective assessment of the comprehensive research capabilities of countries, institutions, and authors. The g-index requires that “the total number of citations for the top g papers by citation frequency be at least *g*^*2*^.” Compared to the h-index, the g-index places greater weight on highly cited papers and is suitable for evaluating entities with significant differences in output scale (such as countries or institutions with varying publication volumes). *k* = 25 is the default setting in CiteSpace; in preliminary tests of this dataset, the modularity *Q*-value of the network was highest *when k =* 25;

#### 2.3.3. Network pruning

Pathfinder algorithm+slice-based network pruning. The Pathfinder algorithm reduces network density by approximately 60% without altering the network’s basic topology by retaining key connections that satisfy the triangle inequality and eliminating redundant, weak connections; slice-based network pruning retains the strongest connections within each time slice, ensuring consistent comparability across time. This combination is a commonly used optimization approach in the field of CiteSpace visualization.

#### 2.3.4. Visualization modes

Static clustering view, timeline view, time-zone view, and emerging term detection are all enabled to provide a multidimensional analysis of the evolution of domain themes.

### 2.4. Scope of the analysis

To enhance the depth of the research and address the limitations of analyzing publication volume alone, this study employs the following multidimensional analysis modules:

Collaboration Network Analysis: Author collaboration networks, country collaboration networks (to quantify the intensity of international collaboration), and research institution collaboration networks.

Disciplinary Distribution Analysis: Classifying and statistically analyzing the disciplines to which the literature belongs based on the WoSCC classification, and mapping the disciplinary distribution.

Keyword Analysis: Keyword co-occurrence, clustering, timelines, and time-zone views.

Frontier Detection: Detection of emerging terms to identify shifts in research hotspots across different phases.

## 3. Results

### 3.1. Trends in annual document output

An annual publication comparison chart was created based on data from both WoSCC (2006–2026, 601 articles) and PubMed (1984–2024, 960 articles) (Fig. 2). There are notable differences in the coverage characteristics of the 2 databases: PubMed includes scattered early publications from 1984 to 2005, with a rapid increase in publication volume after 2007; WoSCC has seen a steady increase in the number of publications since 2006 and has maintained a high and stable output in recent years.

**Figure 2.**
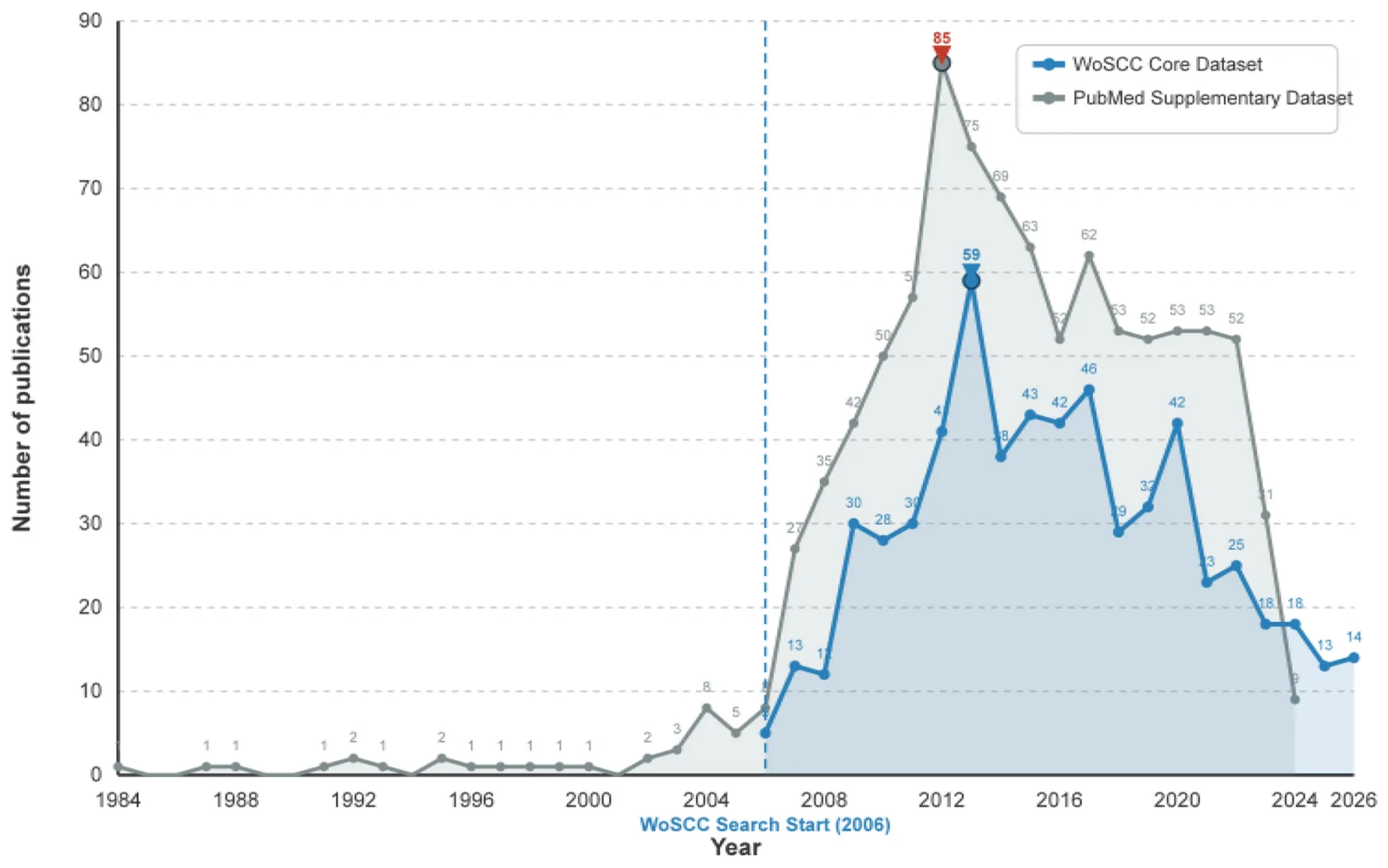
Annual publication trends comparison between WoSCC and PubMed. WoSCC = Web of Science Core Collection.

The total number of global publications has shown a long-term upward trend and can be divided into 3 phases: The Initial Exploration Phase (1984–2006): Annual publication volumes were extremely low, with only a few teams conducting preliminary isolation and characterization of amniotic fluid stem cells; research was limited to describing the basic biological characteristics of the cells. Rapid Growth Phase (2007–2013): Interest in the field grew rapidly, with multiple teams worldwide conducting basic experiments such as multi-lineage differentiation and in vitro culture, leading to a year-on-year increase in the number of publications. Stabilization and Deepening Phase (2014–present): Annual publication volumes have remained at a high level with slight fluctuations; research is no longer limited to basic cellular characteristics but has gradually expanded to multi-level topic (TS) such as differentiation mechanisms, paracrine effects, disease models, and regenerative therapies. Time-series data merely objectively reflect changes in academic research interest and trends in publication TSs; they cannot directly validate the actual effectiveness of clinical translation technologies.

### 3.2. Analysis of research collaboration networks

#### 3.2.1. Author collaboration networks

A collaboration network map of authors generated using CiteSpace 6.2.R4 (Fig. 3) shows that a total of 544 researchers in this field have published papers, with 970 pairs of collaborative relationships among authors; the network density is only 0.0066, indicating a relatively low overall level of collaboration. The largest connected subnetwork comprises 315 nodes, accounting for 57% of all authors, indicating that while a certain academic community exists within the field, cross-team collaboration remains insufficient.

**Figure 3.**
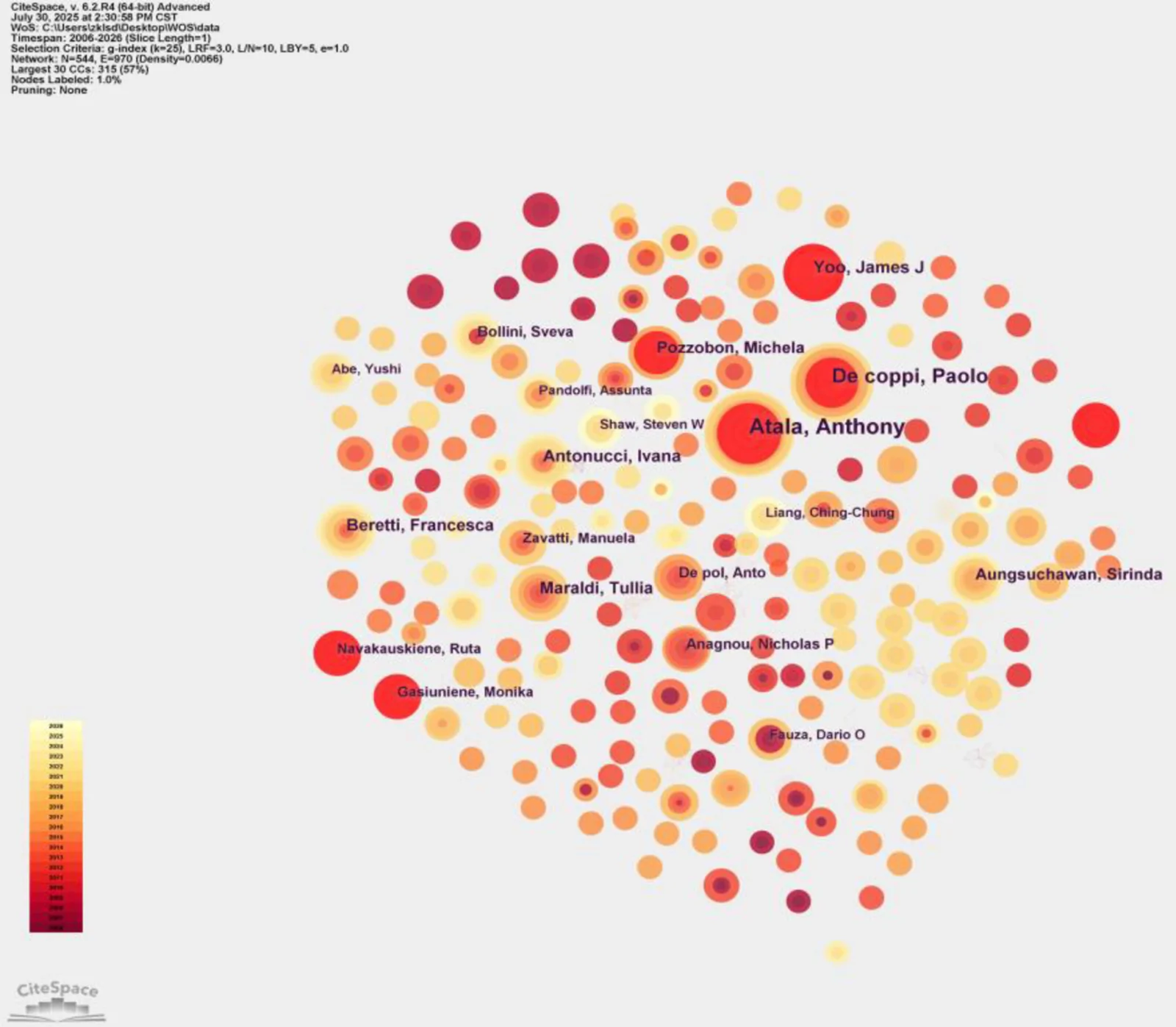
Author collaboration network co-occurrence graph.

In terms of leading authors, Atala (28 publications, 2007), De Coppi (22 publications, 2008), Yoo (12 publications, 2011), and Antonucci (12 publications, 2009) constitute the core group of researchers in this field. The temporal distribution shows that the field underwent a pioneering phase from 2006 to 2010, a period of rapid growth from 2011 to 2015, and a phase of diversified expansion from 2016 to the present. In terms of regional distribution, Italian scholars form the most concentrated collaborative cluster, while the U.S.–U.K. core axis serves as the field’s knowledge engine. Scholars from Southeast Asia and the Baltic region began to become active in the late 2010s, reflecting the gradual diffusion of the field’s research focus (see Table 1 for the top 10 authors by number of publications).

**Table 1:** Top 10 authors by number of published papers.

Rank	Author name	Number of publications	Start yr	Centrality	Country/institution
1	Atala, Anthony	28	2007	0.03	United States/ Wake Forest University
2	De Coppi, Paolo	22	2008	0.03	Italy/ United Kingdom
3	Yoo, James J	12	2011	0	United States/ Wake Forest University
4	Antonucci, Ivana	12	2009	0.02	Italy
5	Maraldi, Tullia	11	2012	0	Italy
6	Beretti, Francesca	11	2013	0	Italy
7	Pozzobon, Michela	10	2011	0	Italy
8	Aungsuchawan, Sirinda	10	2016	0	Thailand
9	Anagnou, Nicholas P	8	2007	0	Greece
10	De Pol, Anto	8	2012	0	Italy

The table lists the publication count, first-published year, betweenness centrality, and corresponding country/institution for each core author.

#### 3.2.2. National cooperation networks and the intensity of international cooperation

A collaborative network map was constructed using countries and regions as nodes (Fig.4). The map includes a total of 49 country nodes and 146 international collaboration links, with a network density of 0.1241. All nodes are connected within the same collaborative network (the largest subnetwork accounts for 100%), indicating that a global academic collaboration system has been established in this field. The top 5 countries by publication volume are the United States (177 papers), Italy (132 papers), China (75 papers), the United Kingdom (59 papers), and Taiwan, China (44 papers). These countries collectively published 487 papers, accounting for more than 60% of the total output across all countries, and constitute the main driving force behind research in this field.

**Figure 4.**
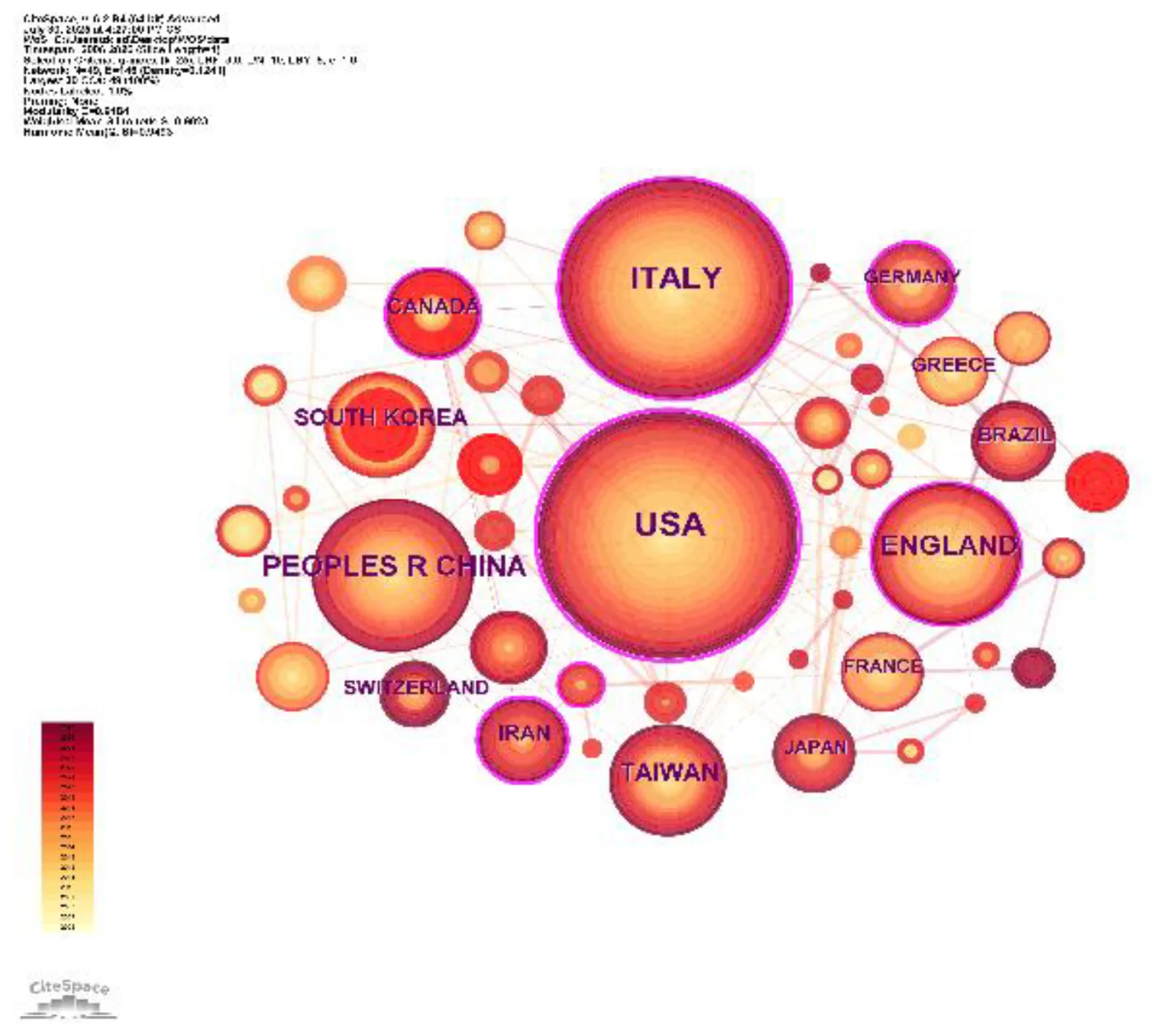
Network Map of National Cooperation.

An analysis of intermediary centrality reveals that the United States (0.53) and Italy (0.38) are the most critical hub nodes in this collaborative network. Not only do these 2 countries lead in research output, but they also play a central intermediary role in cross-regional collaboration. Iran (0.21), the United Kingdom (0.16), and Canada (0.15) also exhibit relatively high centrality, playing a significant bridging role despite their moderate research output. In contrast, China (0.02), Taiwan, China (0.02), and Brazil (0.02), despite their substantial publication output, have relatively narrow channels for international collaboration and limited bridging functions.

The results of the cluster analysis show that the modularity coefficient *Q* = 0.9184, indicating an extremely clear clustering pattern among countries. The weighted average silhouette coefficient *S* = 0.9823 suggests that countries within each cluster are highly homogeneous in terms of research directions and cooperation models. Countries can generally be divided into several cooperation blocs based on geopolitical and cultural factors, including the North America–UK axis, the Mediterranean cluster, the East Asia cluster, the Middle East cluster, and the Latin America cluster, reflecting that geographical proximity and linguistic and cultural commonalities continue to play a significant role in international cooperation in this field. (See Table 2 for the top 10 countries by number of publications.)

**Table 2:** Top 10 countries in terms of the number of published literature.

Rank	Country/Region	Number of publications	Start yr	Centrality
1	USA	177	2006	0.53
2	ITALY	132	2006	0.38
3	PEOPLE’S REPUBLIC OF CHINA	75	2006	0.02
4	ENGLAND	59	2007	0.16
5	TAIWAN	44	2007	0.02
6	SOUTH KOREA	32	2007	0
7	CANADA	23	2009	0.15
8	IRAN	22	2007	0.21
9	BRAZIL	21	2012	0.02
10	GREECE	18	2006	0

Betweenness centrality reflects the bridging capacity of international research collaboration.

#### 3.2.3. Network of collaborating research institutions

A collaboration network map was constructed using research institutions as nodes (Fig. 5). The map comprises a total of 368 institutional nodes and 827 collaboration links, with a network density of 0.0122. The largest connected subnetwork encompasses 302 institutions (accounting for 82%), indicating that research institutions in this field have formed a relatively close-knit academic collaboration system. The top 5 institutions by number of publications are University College London (46), the University of London (46), Wake Forest University (34), Chang Gung University (24), and Great Ormond Street Hospital (24).

**Figure 5.**
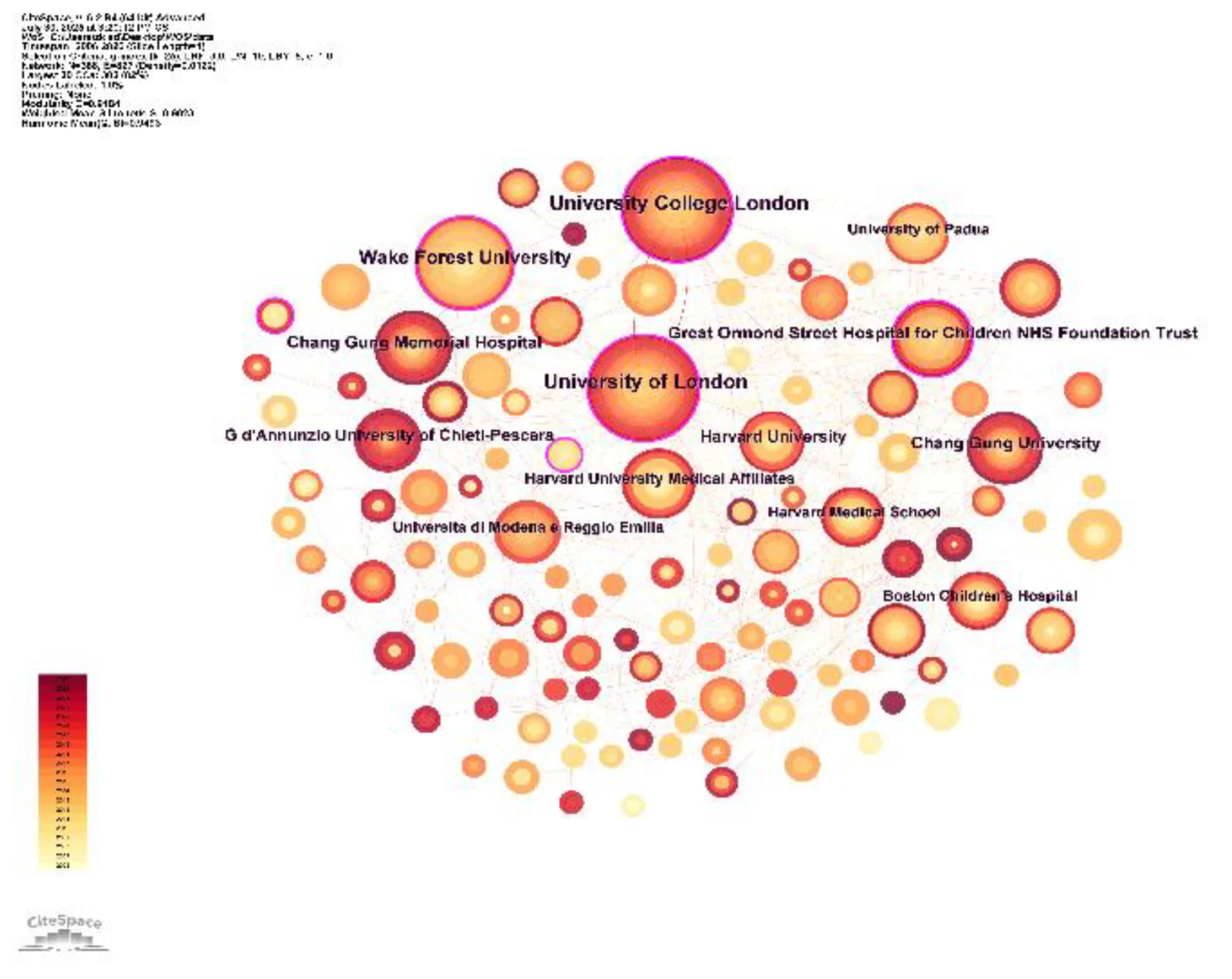
Collaboration network map of research institutions.

Analysis of intermediary centrality reveals that Wake Forest University (0.18) and University College London (0.15) are the most critical hub nodes in this network, serving as key intermediaries connecting institutions across multiple regions in Europe, the United States, and Asia. Furthermore, although the Georgia Institute of Technology (centrality 0.14, 4 publications) and the National University of Singapore (centrality 0.13, 4 publications) have limited output, they play a significant role in connecting across clusters and are high-potential nodes in the international collaboration network.

The results of the cluster analysis show that the modularity (*Q*) is 0.9184, indicating an extremely distinct geographic-administrative clustering structure among institutions; the weighted average silhouette coefficient (S) is 0.9823, suggesting that institutions within each cluster are highly homogeneous in terms of research focus. The major clusters include the London cluster (UCL, Great Ormond Street Hospital), the Wake Forest cluster (Wake Forest University), the Chang Gung cluster (Taiwan’s Chang Gung system), the Harvard-Boston cluster, and the multi-institution cluster in Italy, reflecting how geographic proximity and academic traditions have jointly shaped the current collaborative landscape in this field. (See Table 3 for the top 10 research institutions by number of publications.)

**Table 3:** Top 10 Research institutions by number of published papers.

Rank	Institution name	Number of publications	Start yr	Centrality	Country/Region
1	University College London	46	2008	0.15	United Kingdom
2	University of London	46	2008	0.15	United Kingdom
3	Wake Forest University	34	2007	0.18	United States
4	Chang Gung University	24	2007	0.07	Taiwan, China
5	Great Ormond Street Hospital for Children NHS Foundation Trust	24	2007	0.1	United Kingdom
6	Chang Gung Memorial Hospital	23	2007	0.08	Taiwan, China
7	G d’Annunzio University of Chieti-Pescara	20	2009	0.04	Italy
8	Harvard University	19	2006	0.04	United States
9	Harvard University Medical Affiliates	18	2006	0.04	United States
10	Universita di Modena e Reggio Emilia	16	2012	0.02	Italy

Data include publication count, starting year, betweenness centrality, and affiliated region.

### 3.3. Keyword co-occurrence and cluster analysis

#### 3.3.1. Keyword co-occurrence map

A co-occurrence network graph was constructed using keywords as nodes (Fig. 6). The graph contains a total of 490 keyword nodes and 2957 co-occurrence links, with a network density of 0.0247. All nodes are connected within the same network, indicating that research TSs in this field exhibit good internal coherence. The top 5 keywords by frequency are “differentiation” (159 occurrences), “amniotic fluid” (158 occurrences), “stem cells” (127 occurrences), “mesenchymal stem cells” (109 occurrences), and “expression” (98 occurrences). These keywords encompass the core elements of hAFSC research cell source (amniotic fluid), cell type (stem cells/ mesenchymal stem cells), core biological function (differentiation), and the research paradigm for molecular mechanisms (expression) and constitute the basic framework of the field’s knowledge system.

**Figure 6.**
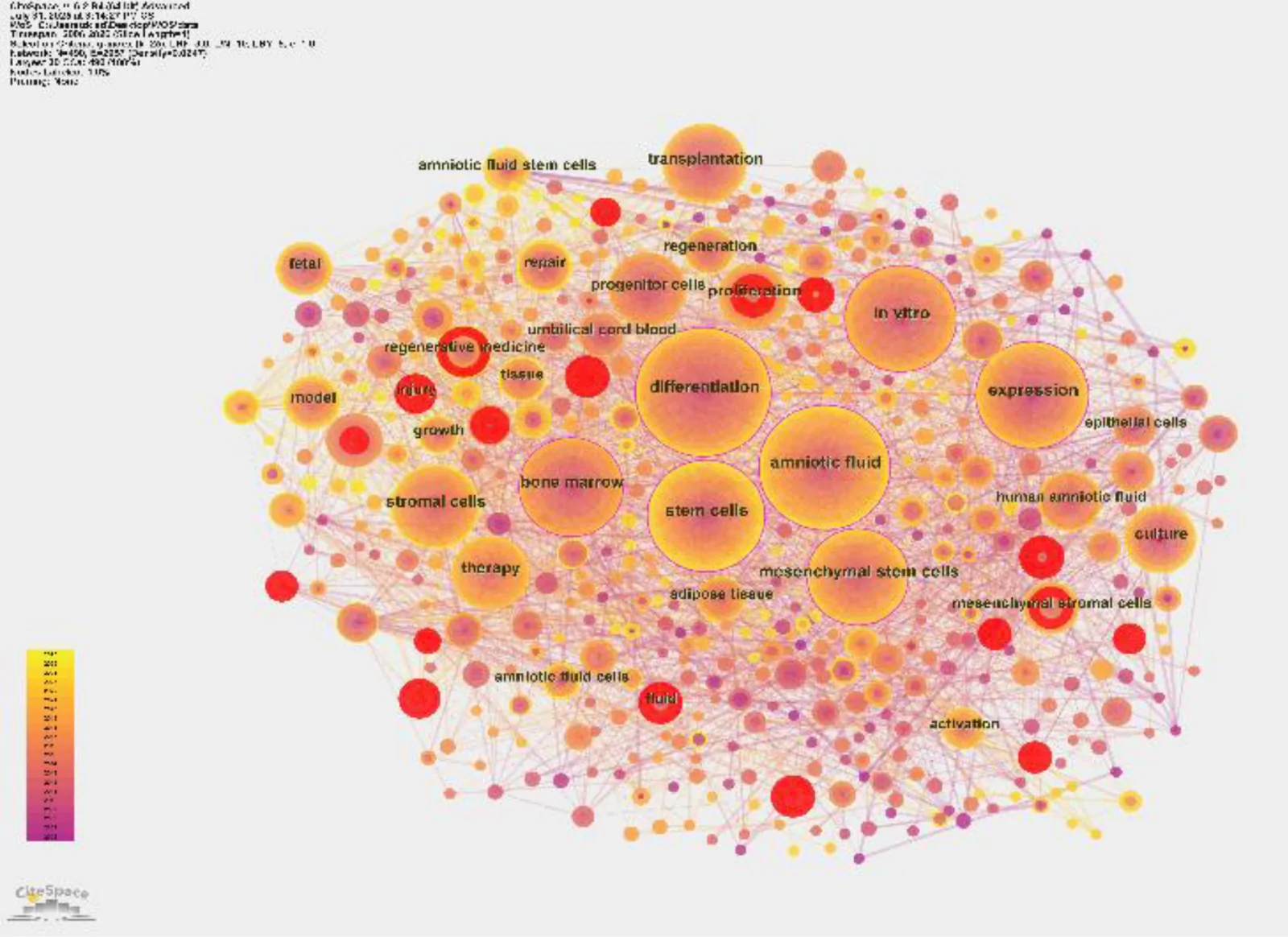
Keyword Co-occurrence Map.

An analysis of intermediary centrality revealed that “stem cells” (0.17), “expression” (0.14), “in vitro” (0.13), “amniotic fluid” (0.11), and “mesenchymal stem cells” (0.11) had the highest centrality values, indicating that these concepts play the most critical hub roles in connecting different research directions. It is worth noting that there is a high degree of overlap between high-frequency keywords and high-centrality keywords, reflecting a strong alignment between the research hotspots in this field and the core of its knowledge structure.

A temporal evolution analysis shows that core keywords such as “differentiation,” “amniotic fluid,” “mesenchymal stem cells,” and “in vitro” have remained active since the field’s inception in 2006, forming the main research thread throughout the entire period. Between 2009 and 2013, keywords such as “transplantation,” “therapy,” “regenerative medicine,” “osteogenic differentiation,” and “neural differentiation” emerged in large numbers, marking a comprehensive expansion of research TSs from basic cell biology to specific clinical applications and multi-directional differentiation lineages. Since 2017, the concentrated appearance of keywords such as “extracellular vesicles” (2020, 14 occurrences), “exosomes” (2017, 11 occurrences), oxidative stress (2018, 7 occurrences), and DNA methylation (2017, 4 occurrences) has reflected a shift in research focus from the traditional “cell replacement” paradigm toward new mechanisms such as “paracrine/exosome-mediated” and “epigenetic regulation.” (See Table 4 for the top 10 keywords.)

**Table 4:** The top 10 keywords with the highest occurrence frequency.

Rank	Keyword	Frequency	Centrality	Start yr	Research dimension
1	Differentiation	159	0.1	2006	Basic biological functions
2	Amniotic fluid	158	0.11	2006	Cell source
3	Stem cells	127	0.17	2007	Core research topics
4	Mesenchymal stem cells	109	0.11	2006	Cell type localization
5	Expression	98	0.14	2006	Molecular mechanism research
6	In vitro	97	0.13	2006	Experimental methodology
7	Bone marrow	90	0.1	2006	Comparison of stem cell Sources
8	Stromal cells	73	0.06	2008	Cell subtypes
9	Transplantation	64	0.05	2009	Application directions
10	Therapy	55	0.09	2010	Clinical translation

“Starting year” represents the year when the keyword firstly emerged in the dataset.

#### 3.3.2. Keyword cluster analysis

A keyword clustering analysis based on CiteSpace identified a total of 36 clusters, including 10 major clusters (Clusters 0–9) directly related to hAFSC research, as well as Clusters 10 to 13. These clusters collectively encompassed 490 keywords, 2957 network connections, and a network density of 0.0247 (Fig. 7). The *Q*-value for the clustering module was 0.3392, and the weighted average *S*-value was 0.6897, indicating that the clustering structure is relatively clear and the internal consistency is acceptable.

**Figure 7.**
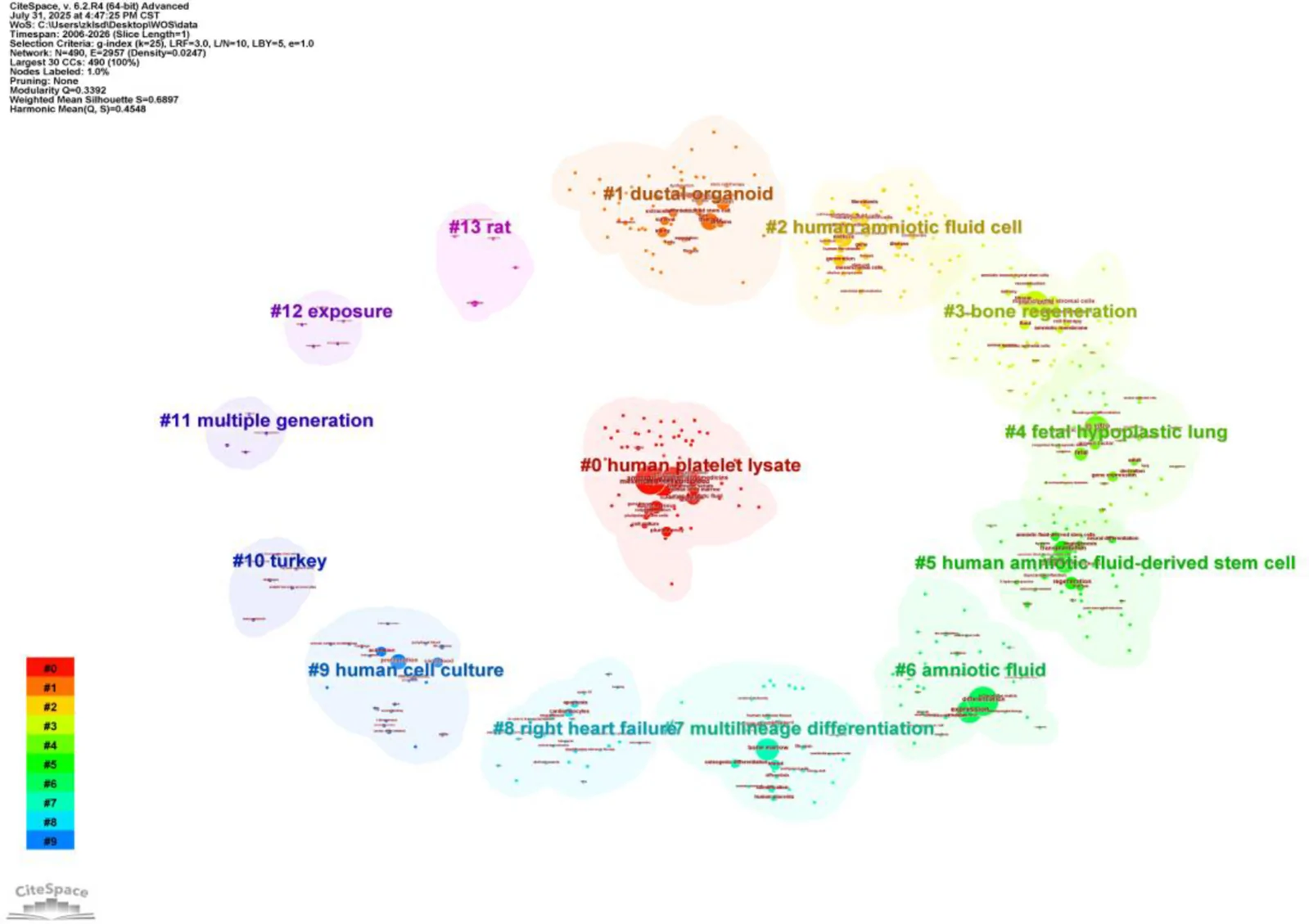
Keyword Clustering Map.

When sorted by average year, the 10 major clusters exhibit a clear chronological sequence: The earliest clusters to form were #7 Multilineage Differentiation (2009, outline value 0.787) and #2 Human Amniotic Fluid Cells (2010, outline value 0.690), representing the core issues in the early stages of this field cell identification and verification of multilineage differentiation potential. Subsequently, #4 Fetal Hydrocephalus (2012), #5 Neural Differentiation (2013), #0 Human Mesenchymal Stem Cells and Platelet Lysate (2014), #8 Right Heart Failure and Pulmonary Hypertension (2014), and #9 Cartilage Differentiation (2014) were successively formed, marking a comprehensive expansion of research themes from basic biology toward specific applications (fetal diseases, neural differentiation, cardiovascular diseases, and cartilage repair). The most recently formed cluster is #1, “Tubular Organoids and Extracellular Vesicles” (2018, clustering score 0.639), representing the cutting-edge directions in this field organoid construction and research on exosome-mediated paracrine mechanisms.

Based on the profile values, #7 Multidirectional Differentiation (0.787), #8 Right Heart Failure and Pulmonary Hypertension (0.796), and #9 Human Cell Culture and Cartilage Differentiation (0.749) exhibit the highest internal consistency, representing the three most robust knowledge modules in this field. The cluster with the lowest outline value is #6, “Amniotic Fluid Microenvironment and 3D Culture” (0.552), whose themes are relatively dispersed, covering multiple technical areas such as microfluidics, 3D dynamic culture, and proteomics.

### 3.4. Temporal evolution of research topics: timeline and time zone views

To further illustrate the longitudinal evolution of research hotspots, this study constructed a keyword timeline view and a time-zone view. As shown in the timeline view (Fig. 8), the 10 major clusters span the period from 2006 to 2026. The earliest clusters to form were #7 Multidirectional Differentiation (2006–2015) and #2 Human Amniotic Fluid Cells (2006–2016), representing core TSs from the field’s early stages; the latest cluster to form was #1 Ductal Organoids and Extracellular Vesicles (2016–2026), representing current cutting-edge directions. In terms of duration, #3 Bone Regeneration (2009–2021), #4 Fetal Diseases (2007–2021), and #6 Amniotic Fluid Microenvironment (2010–2021) are the longest-standing research themes in this field, indicating that bone tissue engineering, the treatment of congenital fetal diseases, and research on the amniotic fluid-derived microenvironment constitute the most enduring knowledge modules in this field.

**Figure 8.**
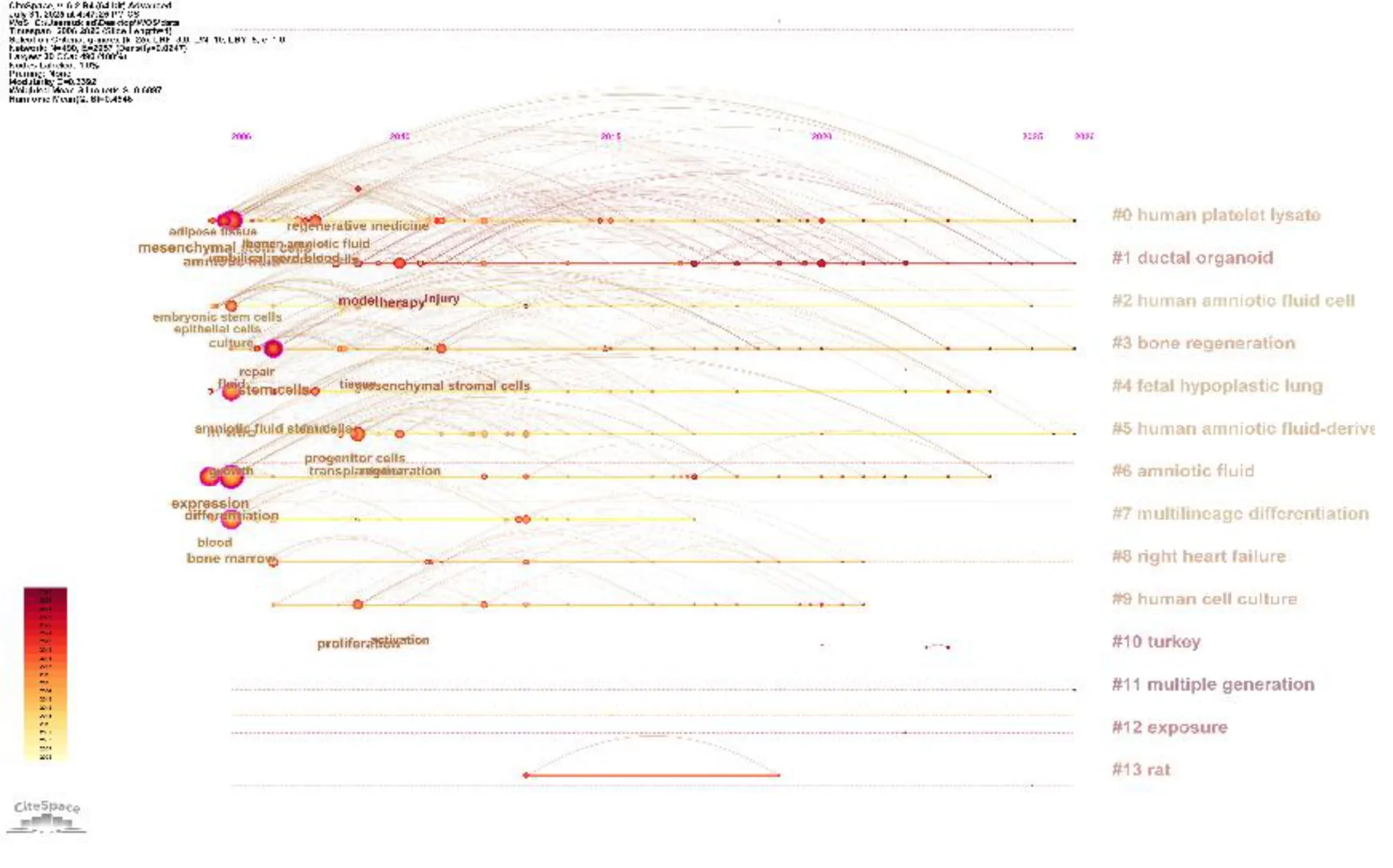
Keyword Timeline View.

The time zone view provides a more detailed breakdown of the distribution of keywords that first appeared in each year (Fig. 9). In 2006, core concepts such as “differentiation,” “amniotic fluid,” “mesenchymal stem cells,” “expression,” “in vitro,” and “bone marrow” emerged in large numbers, forming the foundational framework of the field’s knowledge system. Between 2009 and 2010, keywords such as “transplantation,” “therapy,” “regenerative medicine,” “myocardial infarction,” and “angiogenesis” emerged in large numbers, marking a comprehensive shift in research focus from basic cell biology toward clinical applications. Between 2017 and 2020, the concentrated emergence of keywords such as “exosomes,” “extracellular vesicles,” “DNA methylation,” “oxidative stress,” and “inflammation” reflected a shift in research focus from the traditional “cell replacement” paradigm toward new mechanistic paradigms such as “paracrine/exosome-mediated” and “epigenetic regulation.” Notably, the recent emergence of keywords such as “myelomeningocele” (2025) and “mechanism” (2026) suggests that, while the field continues to expand its clinical indications, it is also returning to a reexamination of underlying mechanisms.

**Figure 9.**
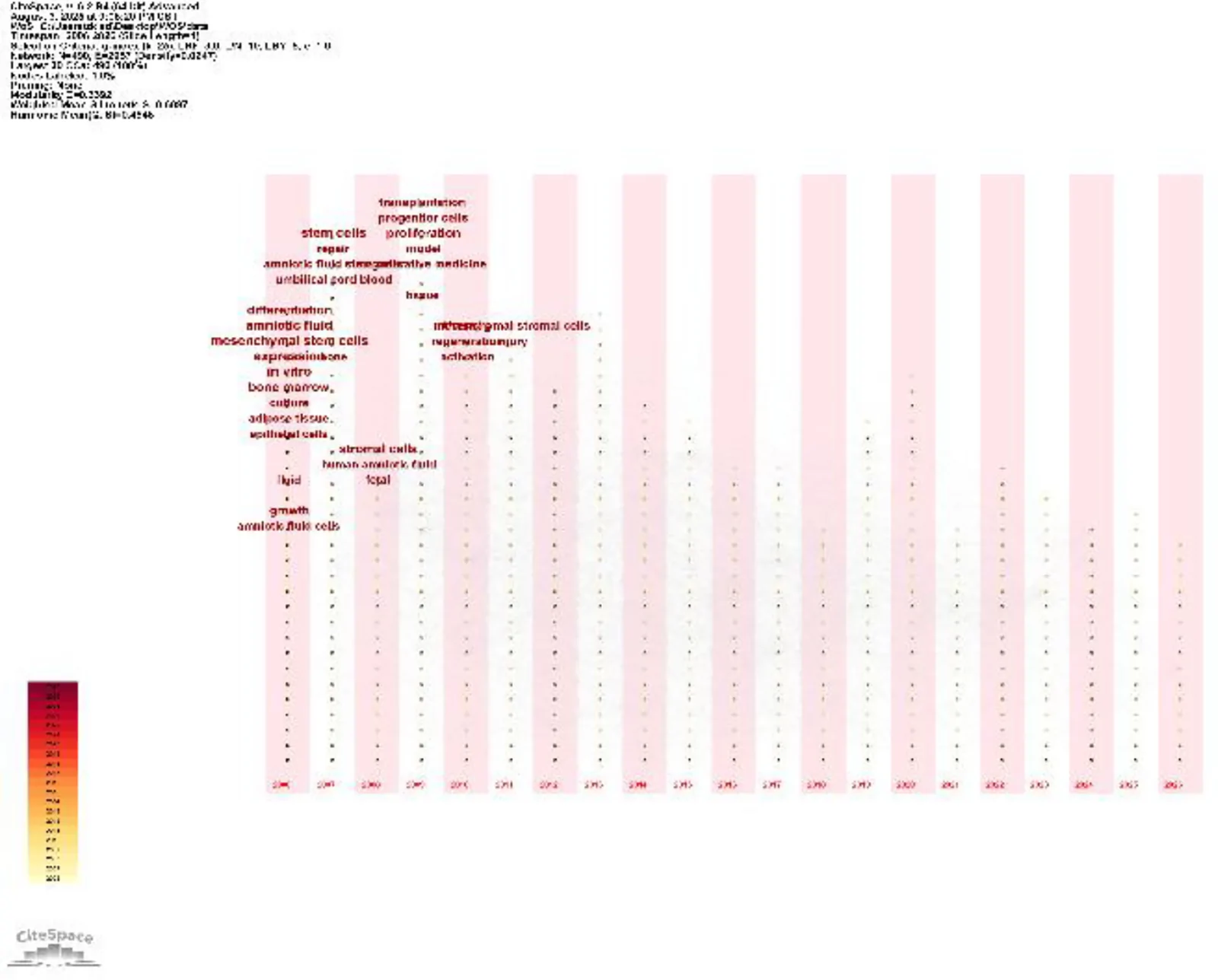
Keyword TimeZone View. Top 19 Keywords with the Strongest Citation Bursts.

### 3.5. Analysis of emerging terms at the cutting edge

The salience analysis identified a total of 19 keywords with significant salience scores (Fig. 10). Ranked by salience score, the top five were “extracellular vesicles” (7.37), “growth factor” (6.09), “induction” (5.26), “injury” (4.66), and “culture” (4.13).

**Figure 10.**
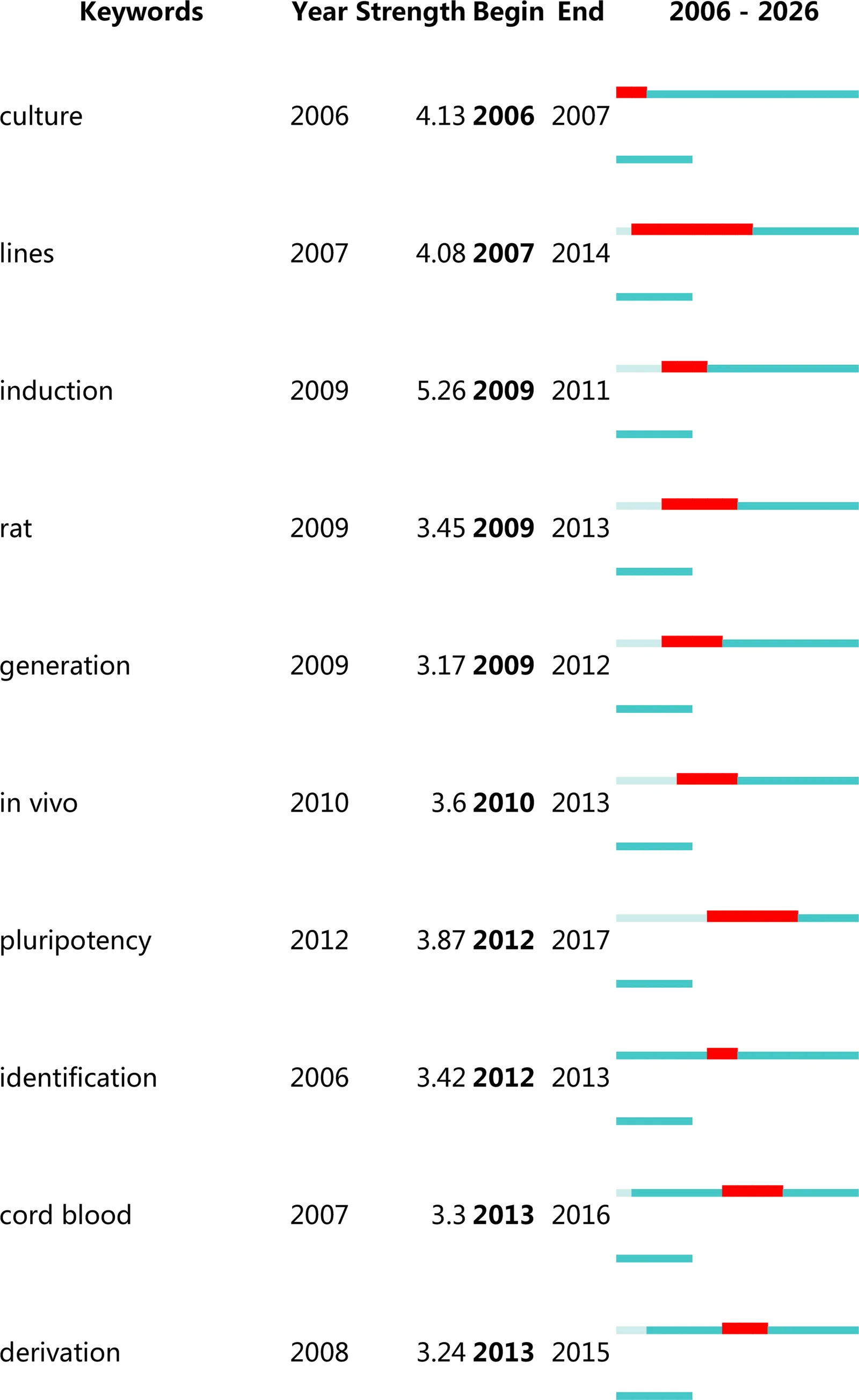
Keyword bursts.

In terms of temporal distribution, the emerging terms exhibit a clear 3-stage evolutionary pattern. In the first stage (2006–2008), “culture” (2006–2007) and “lines” (2007–2014) emerged first, reflecting the fundamental needs for cell culture methods and the establishment of cell lines during the field’s early stages. In the second phase (2009–2017), the keywords “induction” (2009–2011, intensity 5.26), “rat” (2009–2013), “in vivo” (2010–2013), pluripotency (2012–2017), proliferation (2014–2016), and growth factor (2017–2019, intensity 6.09) successively emerged, marking a systematic expansion of research themes from basic methodology to induced differentiation, validation using animal models, pluripotency assessment, and the exploration of molecular mechanisms. In the third phase (2018–2026), the terms “extracellular vesicles” (2020–2024, intensity 7.37), “exosome” (2020–2026), “injury” (2018–2026), and “mice” (2021–2023) emerged prominently, representing the most active current research frontiers exosome/extracellular vesicle-mediated paracrine mechanisms, targeted therapies for specific types of injury, and the application of mouse models.

It is worth noting that “extracellular vesicles” ranked first with an emergence intensity of 7.37, far surpassing other keywords, signaling a dramatic shift in the research paradigm in this field since 2020 a transition from the traditional “cell replacement” paradigm to the “paracrine/exosome-mediated microenvironment regulation” paradigm. Meanwhile, the emergence windows for the keywords “injury” (2018–2026) and “exosm” (2020–2026) extend through the data collection cutoff year (2026), indicating that these 2 research directions are currently still gaining momentum and represent the most noteworthy emerging growth areas in the field.

## 4. Discussion

### 4.1. Methodological advantages and chronological patterns in the literature

This study established a dual-database search framework centered on WoSCC and supplemented by PubMed, fully addressing the requirements of bibliometric standards. From WoSCC, 601 original research articles published between 2006 and 2026 were selected as the core dataset, and a comprehensive, multidimensional network map was constructed using complete citation data. From PubMed, 960 articles published between 1984 and 2024 were retained solely for comparison of publication trends during the field’s nascent stage. The 2 databases complement each other’s strengths, addressing the lack of citation metadata in a single database while ensuring complete coverage of early-period literature. Both databases fully disclose standardized search queries, and the literature screening strictly adheres to the PRISMA 2020 guidelines, ensuring the search process is fully reproducible.

Based on the annual publication statistics from the 2 databases, the total number of publications in the field of hAFSCs from 1984 to 2026 has continued to rise. This period can be broadly divided into 3 developmental stages: 1984 to 2006 was the nascent exploration phase, during which only a small number of teams conducted preliminary studies on cell isolation and basic characterization. From 2007 to 2013, the field entered a period of rapid growth, as a series of pioneering achievements by the teams led by Atala and De Coppi spurred a surge of global research teams, resulting in a rapid year-on-year increase in the number of publications. From 2014 to the present, the field has entered a period of stable deepening, with annual publication numbers maintaining a high level while fluctuating slightly, and the research focus has gradually expanded from purely cellular biological characterization to multi-level directions such as molecular regulatory mechanisms, animal disease models, and regenerative translation. It should be noted that this study conducts only descriptive statistics on publication trends; it can objectively reflect research preferences within the academic community but cannot directly validate the actual clinical translational efficacy of hAFSCs. The entire text avoids making absolute clinical inferences. As research continues to advance, existing findings confirm that hAFSCs possess broad application potential in fields such as tissue engineering, cell therapy, disease model construction, and drug screening. Current research is primarily focused on in-depth exploration of stem cell differentiation regulation, signaling pathways, immunomodulation, and clinical translation strategies.^[9–11]^

### 4.2. The global landscape of scientific research collaboration

#### 4.2.1. Author collaboration network

The author collaboration network comprises 544 nodes and 970 collaboration links, with a network density of only 0.0066, reflecting insufficient cross-team communication among global researchers and a pronounced fragmentation of research. Atala, De Coppi, and Yoo James J. are the 3 leading scholars in this field; their teams’ work has laid the foundation for hAFSC isolation, tissue engineering, and preclinical research. Italian scholars form the most closely knit local research cluster, while Europe and the United States constitute the core academic hubs of the field. Relevant teams in Southeast Asia and the Baltic region have gradually become involved in this field of research since 2010.

#### 4.2.2. National collaboration network

The National Collaboration Network spans 49 countries, with all nodes interconnected to form a global collaboration system. The United States (177 papers, betweenness centrality 0.53) and Italy (132 papers, betweenness centrality 0.38) form a “dual-core engine” of international collaboration, ranking among the top in both publication output and cross-regional bridging capacity. China ranks third globally with 75 publications, but its betweenness centrality is only 0.02, exhibiting a pattern of “high productivity but low international connectivity.” The proportion of domestic independent research is relatively high, while multilateral collaborative research is relatively scarce. Clustering results (Modularity *Q* = 0.9184, weighted silhouette *S* = 0.9823) show that countries have formed distinct collaboration clusters such as North America, the Mediterranean, East Asia, and the Middle East based on geography and language, indicating that geography and culture remain key factors influencing international collaboration.

#### 4.2.3. Institutional collaboration network

The institutional network comprises 368 nodes, with University College London (UCL), Wake Forest University, and the Chang Gung University system ranking in the top 3 for publication output, forming a tripolar dominance pattern centered on “London, North Carolina, and Taiwan, China.” Among these, Wake Forest University (0.18) and UCL (0.15) have the highest intermediary centrality, serving as core hubs connecting research institutions in Europe, the United States, and Asia. While Georgia Institute of Technology and the National University of Singapore have limited total publication volumes, their role in facilitating cross-cluster connections is prominent, suggesting that Asian research institutions still have room to expand their international collaborations.

### 4.3. Evolution of research themes and shifts in research focus

By integrating multidimensional maps that incorporate keyword co-occurrence, clustering, timelines, time zones, and emerging terms, it is possible to comprehensively trace the progressive development of the hAFSCs field, from basic in vitro experiments to cell-free regenerative therapies.

Keyword co-occurrence analysis reveals that “differentiation” (159 occurrences), “amniotic fluid” (158 occurrences), “stem cells” (127 occurrences), and “mesenchymal stem cells” (109 occurrences) are the top 4 core keywords by frequency, forming the “cell source cell type” core biological functions molecular mechanisms, forming a comprehensive research framework. There is a high degree of overlap between high-frequency terms and high-intermediate central terms, reflecting the highly focused research agenda in this field. Current research continues to advance in core areas such as cell differentiation, in vitro culture, tissue engineering, and cell therapy, while simultaneously exploring the application value of hAFSCs in reproductive medicine and organ damage repair.^[12–15]^

The temporal patterns of keyword clusters are distinct: #7 Multilineage Differentiation (2009) and #2 Human Amniotic Fluid Cells (2010) represent clusters of early-stage basic research in the field, focusing on cell identification and verification of multipotency. From 2012 to 2014, clusters such as #4 Fetal Diseases, #5 Neural Differentiation, #3 Bone Regeneration, and #8 Cardiopulmonary Injury Repair emerged in rapid succession, with research extending to multi-organ directed differentiation and animal models of fetal congenital diseases. The #1 Extracellular Vesicles and Organoids cluster (2018) represents the latest research area, reflecting current cutting-edge exploration directions.

Analysis of emerging terms clearly reveals a paradigm shift in the field: “extracellular vesicles” had an emergence intensity of 7.37, the highest value in the entire network, significantly higher than “growth factor” (6.09) and “induction” (5.26), indicating that after 2020, the focus of academic research shifted from whole-cell stem cell transplantation to exosome-mediated paracrine cell-free therapies. Extracellular vesicles derived from amniotic fluid can exert anti-inflammatory and regenerative effects by regulating the microenvironment, thereby circumventing the immunological and oncogenic risks associated with live cell transplantation. The emergence cycle of “injury” spans from 2018 to 2026, reflecting an ongoing research focus on precision repair models for various organ injuries. In the temporal view, the simultaneous emergence of new terms such as “myelomeningocele” (2025) and “mechanism” (2026) suggests that, while the field is expanding into new indications such as fetal surgery, it is also returning to an in-depth exploration of underlying molecular regulatory mechanisms.^[16–18]^

### 4.4. Limitations of the study

First, databases such as Scopus and Embase were not included, resulting in a bias toward English-language literature and the exclusion of non-English literature. Second, WoSCC only includes original articles, so important literature types such as reviews and clinical trials were not included in the analysis. Third, the PubMed search was limited to May 2024, which does not align with the WoSCC time window; this discrepancy must be taken into account when making comparisons. Fourth, ambiguities in institutional names (e.g., UCL and the University of London appearing with the same frequency) and fragmentation in the Harvard system may lead to an underestimation of some institutions’ influence; future studies could incorporate name disambiguation algorithms to address this; Fifth, meta-analyses can only describe publication patterns and cannot assess research quality or clinical value.

## 5. Conclusions and outlook

### 5.1. Conclusions

This study employed a standardized search protocol using 2 databases, and the search process can be fully replicated. The number of publications on hAFSCs showed continuous growth from 1984 to 2026, which can be divided into 3 phases based on research depth: the embryonic phase, the rapid growth phase, and the stabilization and deepening phase. The WoSCC and PubMed datasets complement each other chronologically.

Global scientific research collaboration has taken on a “U.S.-Italy dual-core, 3-polar institutional” structure, with top universities and affiliated hospitals in Europe and the United States serving as the primary sources of output, while China ranks third globally in the number of published papers. Its role as a hub for international collaboration remains relatively weak, and there is significant fragmentation in cross-team and cross-regional research.

The field’s knowledge system is logically coherent, with the multidirectional differentiation of stem cells serving as the central thread running throughout. The research timeline is clear, progressing gradually from early basic cellular characterization to multi-tissue differentiation and preclinical models of fetal diseases, with exosomes and organoids emerging as key research focuses in recent years.

Data on emerging keywords confirm a key paradigm shift in the field: interest in traditional live-cell transplantation research has waned, while extracellular vesicle-mediated cell-free tissue repair has become the most prominent frontier, with research increasingly aligned with clinical scenarios of organ injury.

This study is a purely descriptive bibliometric analysis that summarizes field trends based solely on publication activity; it cannot validate the clinical efficacy of hAFSCs.

### 5.2. Outlook

Based on the field characteristics revealed by the network analysis, we propose the following directions for future research aligned with the data: High-output institutions in China should strengthen long-term collaboration with core laboratories in the U.S. and Italy to enhance their status as hubs in the international network; Focus on advancing cell-free translational research in areas such as the standardization of exosome purification, optimization of drug delivery systems, and inflammatory damage in newborns; Conduct interdisciplinary exploration of 3D organoids and epigenetic regulation by integrating biomaterials and bioinformatics; Promote the standardization of experimental protocols for differentiation induction and exosome detection; and Future research metrics should incorporate multiple languages and databases, supplementing indicators such as research funding and per-capita output to provide a more comprehensive picture of the global research landscape.

## Author contributions

**Data curation:** Huanying Lou.

**Formal analysis:** Zheng Wang.

**Methodology:** Shuai Zhang.

**Software:** Feng Wang.

**Conceptualization:** Qiang Feng, Yi Li.

**Writing – original draft:** Huanying Lou.
